# ‘You’re in a new game and you don’t know the rules: Preparing carers to care’

**DOI:** 10.1177/14713012221112242

**Published:** 2022-07-07

**Authors:** Teresa Atkinson, Jennifer Bray, Tracey Williamson

**Affiliations:** The Association for Dementia Studies, 8709 University of Worcester, UK; 1507 Betsi Cadwaladr University Health Board, Bangor, UK; 8709 University of Worcester, UK

**Keywords:** dementia, education, family carers, residential course, training

## Abstract

**Aim:**

Being an informal carer for a person living with dementia can be a demanding role which can have detrimental effects on personal well-being and affect a person’s ability to provide care for their loved one. This evaluation of support courses, offered by a leading UK charity dedicated to dementia family carers, highlights the impact of training to support the caring role.

**Setting:**

Participants completed booklets at the training venue and subsequently online or by post. Interviews with participants took place by telephone. Participants: 84 participants completed booklets containing measures which generated quantitative data whilst 19 family carers participated in qualitative telephone interviews.

**Design:**

A mixed methods approach was taken using booklets of validated measures to capture quantitative data, including capture of demographic information, together with semi-structured interviews conducted by telephone which were recorded, transcribed and subsequently analysed using thematic analysis.

**Results:**

Overall, both the quantitative and qualitative analysis demonstrate that attending the carers support courses had a positive impact on carers with improvements being maintained over time. Outcomes indicated that carers generally remained in a better physical, mental and emotional situation than that experienced before the course.

**Conclusion:**

Being prepared for the trajectory of the caring role when providing care for a person living with dementia can help informal carers to be better prepared, better supported and better informed. Evidence gained from this evaluation demonstrates the impact of the courses and adds to the current weak evidence base relating to dementia courses aimed at preparing carers to care.

## Background

It has been well-documented that there is increasing concern as the numbers of people diagnosed with dementia globally continues to rise. Although there are an estimated 50 million people living with dementia worldwide, this figure is anticipated to triple by 2050 (World Health Organisation [WHO], 2019). The WHO note not only the cost to the public purse but also that dementia is a *‘major cause of disability and dependency among older people and can devastate the lives of affected individuals, their carers’ and families’* (WHO, 2019, p. 3).

The Alzheimer’s Society (2012) report that around two thirds of people with dementia live at home and most people report this would be their preference rather than choosing a move to a care facility if they developed dementia (Alzheimer’s Society, 2016). The number of older adults living at home who require care is set to increase with over half of Local Authorities expecting to see 25% of their population to be over the age of 65 years by 2036 (Office for National Statistics, 2017). With increasing pressures on social care budgets, it is anticipated that the balance of care will continue to fall to informal carers rather than being provided by professional care services (Broese Van Groenou & De Boer, 2016).

Informal carers of people living with dementia constitute a considerable workforce. There are almost 8 million informal carers in the UK, saving the economy £140 billion per year (YouGov, 2019). However, the impact of taking on a caring role can be detrimental for many people. Sixty-five percent of older carers (60–94 years) have a disability or long-term health problems of their own whilst 72% of carers report experiencing mental ill health with 61% reporting physical ill health (Carers Trust, 2015; Carers UK, 2015). Financial pressures are also experienced by 44% of people, with 42% saying the caring role has had an impact on their working life and 29% stating it has impacted on their career (YouGov, 2019). In the UK, there are approximately 700,000 informal carers who care for a person living with dementia with 45% of carers aged 75 years and over.

## The informal caring role

The role of informal carer is often an unanticipated one which is accompanied by additional duties which can have a significant impact on personal wellbeing (Bressan, Visintini, & Palese, 2020, Steenfeldt, 2021; Queluz et al, 2020). This may lead to feelings of emotional and physical exhaustion as a result of sleep deprivation, additional chores and providing practical support. In many cases, informal carers prioritise caring for a family member over their own wellbeing (Quinn, Clare, & Woods, 2015). Guilt about putting their own needs first, combined with feelings of duty and responsibility to care for their loved one can prevent some carers from making use of respite care (Leocadie, Roy, & Rothan-Tondeur, 2018). This is concerning, as carers who take a break from caring are less likely to experience mental ill health than those who do not take a break, and a carer’s ability to care can be affected by poor mental and physical health (*ibid*). Many carers feel lonely or socially isolated as a result of their caring responsibilities (Alzheimer’s Research UK, 2015) whilst some may be part of a ‘sandwich’ generation of carers who care for both elderly relatives whilst still raising their own family (Carers UK, 2019)

Many carers find themselves capitulating into a caring role rather than actively choosing it, so it is perhaps not surprising that carers often have a limited understanding of dementia. Their knowledge is mainly drawn from their own experiences, so they may not be aware of the wider impact of dementia and may not always associate some symptoms with the condition. Although improved knowledge may help carers, many choose not to find out more about dementia and what the future may hold, preferring instead to take each day as it comes (Alzheimer’s Research UK, 2015; Carers UK & Age UK 2015; Dementia Statistics Hub, 2018).

Understanding the dementia ‘journey’ and preparing for its challenges and opportunities can enable carers to strengthen their own resilience, develop strategies and anticipate future needs for both themselves and their loved ones. Research suggests that those who experience more positive aspects of caring benefit from lower levels of depression and anxiety, find meaning and gratification leading to increased morale and improved coping, ultimately reducing the likelihood of residential care for the person living with dementia (Lloyd, Patterson, & Muers, 2016).

## Preparing family carers for their caring role

The needs of those caring for people with dementia is at the heart of work carried out by Dementia Carers Count (DCC). This organisation is founded upon the long and rich history established by the charitable works of the Royal Surgical Aid Society (RSAS) founded in 1862. At its 150th anniversary, RSAS refocused from care home provision to supporting carers of people living with dementia through a range of education-based services.

In 2017, DCC launched a programme of face-to-face courses to inspire those caring for people with dementia to learn, connect and receive practical support to feel empowered in their caring role.

## The courses

In developing a core three-day course for carers, DCC drew on the work of the *Going to Stay at Home Program* which had been implemented in Australia (Gresham, Tsang, Heffernan, & Brodaty, 2014). This was a seven-day residential carer training programme based on a model previously reported by Brodaty and Gresham (1989). The Australian programme had shown a significant reduction in carers’ psychological distress and a delay in admission to residential care for the person living with dementia. Additionally, DCC established a Carer’s Advisory Panel to ensure the courses and their delivery were rooted in the carer experience. This comprised six current or ex-carers of a person living with dementia acting in an advisory role. The Carer’s Advisory Panel was instrumental in co-producing the original three-day residential course which included the following elements:

- Knowledge based sessions.
- Time for reflection.
- Resilience and coping.
- Focuses on carers.
- Self-help – tools to take away (preparing for future challenges).
- Fostering a spirit of resilience and spirit of self-coaching.

The three-day course was based on a residential model with venues carefully chosen to maximise accessibility for carers. Courses were provided face-to-face within a training room located at the venue providing for a maximum of 18 participants supported by two training facilitators. Care was taken to provide high quality accommodation with sufficient space for break out groups, recreation/social spaces and excellent hospitality.

An important element of the core three-day course was to establish a ‘safe space’ where carers were enabled to discuss their feelings and build confidence in a safe and secure environment. Creating the ‘safe space’ enabled carers to feel confident to share their experiences and have their stories heard. The overnight element of the residential stay was important to create a bond between participants as well as to afford them an opportunity for respite.

An iterative ‘feed-forward’ process of course development was established whereby feedback from carers attending the early core courses was utilised by DCC to drive changes to its overall education programme. Subsequently, three further one-day courses were developed as a direct result of such feedback which were tailored and bespoke to address the issues of concern identified by carers.

The first of these, entitled ‘Me, You, Dementia Too’, was based on a request from carers to be accompanied to a course by the person with dementia. This enabled carers to take advantage of the residential course without having to provide care or respite in their absence. Whilst carers undertook the three-day training programme, the person with dementia was supported on a parallel programme provided by Berkshire Young Onset Dementia Group. The second course offer was entitled ‘Home from Home’, being tailored to support carers for whom the person they cared for had made the transition from home care to a residential setting. It is particularly important to recognise that the caring journey and caring experience does not cease once a person with dementia has moved into residential care but that the experiences of caring may change. The third course addressed the needs of those caring for a person with ‘Young Onset Dementia’, defined as someone living with dementia under the age of 65 years.

## The evaluation

The evaluation phase ran from October 2017 to June 2019, during this time 12 courses were delivered: eight core courses; two Me, You, Dementia Too; and one each of the Young Onset Dementia and Home from Home. As part of the evaluation, an initial scoping study was conducted to look at existing UK courses for people caring for a person with dementia and see how the DCC courses fitted within the current landscape of educational support for carers. This was carried out as a desk-based internet search to replicate how a carer may explore the options available to them.

## Method

The evaluation combined quantitative and qualitative data collection methods exploring multiple aspects of carer outcomes. Three types of data were gathered:

### Demographic data

As part of the evaluation, basic demographic data were captured to provide context for participant responses.

### Quantitative data

A series of validated measures were compiled into a single booklet for carers for ease of completion and were made available as a printed version or for online completion. Measures were typically completed prior to commencement of a course and again at three and six-month intervals. Ideally, follow-up was also completed at the twelve-month stage but only participants completing the initial two core courses had sufficient post-course time lapse to enable this to take place. Additionally, the Dementia Knowledge Assessment Scale (DKAS) measure was repeated immediately upon course completion to assess changes in knowledge and understanding of dementia.

The measures contained in the booklet included:

- DKAS – a 19-item measure assessing carers’ knowledge of dementia (Annear et al., 2017).
- Carers – DEMentia Quality Of Life (C-DEMQOL) – a 40-item measure assessing quality of life of people caring for someone with dementia (Brown et al., 2019).
- Kessler Psychological Distress Scale (K10) – a 10-item measure of distress experienced by a person (Kessler et al., 2002).
- Short-Form Health Survey (SF12) – a 12-item measure evaluating personal health (Jenkinson et al., 1997).
- Brief Resilience Scale (BRS) – a 6-item measure to assess a person’s ability to recover from stress (Smith et al., 2008).
- Short Warwick-Edinburgh Mental Wellbeing Scale (SWEMWBS) – a 7-item measure used to monitor mental wellbeing (Tennant et al., 2007).

### Qualitative data

In addition to completion of validated measurements, telephone interviews were carried out with a self-selected sample of carers drawn from across all courses. All carers from all courses were invited and interviews were conducted three months after attendance on a course.

## Data analysis

Descriptive statistics were used to provide an overview of the demographics data relating to the carers, people with dementia and caring relationships. The scores from the outcome measures were analysed using ‘Pairwise t-tests’ to explore potential changes over time. This meant that carers were only included if they had a valid response at both relevant time points.

The interview transcripts were analysed using Thematic Analysis (Braun & Clarke, 2006, 2013). This approach aims to identify themes and patterns of meaning across datasets. It is concerned with context and uses a ‘bottom up’ approach rooted in the words and meaning of the carers to generate a set of themes which is representative of their collective experience.

## Findings

The desk-based review of courses available for carers of people with dementia identified 53 courses in the UK, of which seven were delivered online. For those where more information was available, 35 indicated that they were specifically aimed at family carers while a further nine said that they would be suitable for family carers as well as professionals. Twenty-one courses were one-off sessions lasting less than half a day, while 19 courses comprised multiple short sessions, for example, one 2-hour session per week for 6 weeks. Although these courses may meet the needs of carers in various ways, it is likely that in some cases there may only be enough time to provide an overview of a topic rather than having the opportunity to explore it in any depth. The scoping study therefore indicates that the DCC three-day residential courses specifically focusing on family carers are a unique offer for UK-based carers.

Data from 84 carers completing booklets were included in the evaluation and a total 19 interviews were conducted: nine from participants on a core course; four from a Me, You, Dementia Too course; four from a Home from Home course; and two from a Young Onset Dementia course. Due to the timing of course delivery within the timescales of the evaluation, the number of booklets completed at later time points was reduced (Table 1).

**Table 1.** Number of completed booklets at different time points.

	Pre-course	Post-course	3 months	6 months	12 months
Number of booklets completed	84	80	50	38	8

To provide context for the findings, carers who completed the booklets were aged 20–82 years with a mean age of 56 years. Eighty-three percent of carers were female, and it is acknowledged that consequently the male perspective of caring is likely to be under represented in the results. Reflecting the range of ages, the employment status of the carers was varied with 33% being retired, 31% being employed and 22% not being employed. Only 6% of carers self-identified as disabled, but 31% reported having a long-term health condition. Of those carers with a long-term health condition, 58% had multiple conditions with arthritis/osteoarthritis and high blood pressure being the two most common conditions reported.

The majority of carers reported caring for a parent with dementia (57%), with a further 35% caring for a spouse or partner. Forty-five percent of carers lived with the person they were caring for. Eighty-six percent of the people with dementia being cared for had a diagnosis of dementia, with Alzheimer’s disease accounting for 45% of cases. This was followed by 21% mixed dementia and 17% vascular dementia. Seventy percent of the people with dementia were reported to have at least one other long-term condition with heard conditions, high blood pressure, arthritis/osteoarthritis and diabetes being the most common.

Seventy-seven percent of carers reported being the main carer, with 47% saying they were the only carer. On average, carers had been in a caring role in relation to dementia or other conditions for just under four years and eight months. Sixty percent of carers spend up to seven hours caring per day, with 21% providing care around the clock. Seventy-six percent of carers who provided care for at least 17 hours a day reported doing this seven days a week. Similarly, many carers who care for fewer hours per day do this on almost a daily basis. Other caring responsibilities may have been a factor for some carers, but was outside the remit of the data collected for this study.

The demographics for the 19 interviewees indicate that they are representative of the wider pool of evaluation participants, being similar in terms of average age, split of men and women, employment status and disability. A slightly lower proportion had a long-term health condition than the wider group at 21% compared to 31%. The group was also representative in terms of the diagnoses for the people with dementia being cared for and the caring relationship, suggesting that their views, opinions and perspectives should be typical of the wider group of carers.

Based on the DKAS scores, pairwise t-tests indicated that carers’ knowledge of dementia was improved by attending the courses. It should be noted that as only carers with a valid score at both relevant time points were included in the analysis, the pre-course mean is different for each group, that is, the pre-course mean for the 78 carers who also had a valid post-course score is different to the pre-course mean for the 48 carers included after 3 months. This applies to the results presented in Tables 2–7. There was a significant improvement (increase) in scores immediately after the courses (at the end of the final day), 3 months later and 6 months later (Table 2). Although the improvement was not statistically significant at 12 months, potentially due to the smaller cohort included at this time point, it still showed a positive long-term outcome.

**Table 2.** Dementia knowledge assessment scale scores compared at different time points using pairwise t-tests.

	Post-course	3 months	6 months	12 months
Pre-course	Mean increased from 32.7 to 40.1 (*n* = 78) Significant improvement (*p* < .05, t = −9.41)	Mean increased from 33.0 to 39.5 (*n* = 48) Significant improvement (*p* < .05, t = −6.18)	Mean increased from 33.7 to 40.8 (*n* = 38) Significant improvement (*p* < .05, t = −6.18)	Mean increased from 32.3 to 40.6 (*n* = 7) Improvement (*p* = .06, t = −2.36)

**Table 3.** C-DEMQOL total scores compared at different time points using pairwise t-tests.

	3 months	6 months	12 months
Pre-course	Mean increased from 110.8 to 116.9 (*n* = 41) Significant improvement (*p* = .01, t = −2.93)	Mean increased from 111.9 to 116.1 (*n* = 35) Improvement (*p* = .14, t = −1.50)	Mean increased from 123.5 to 125.7 (*n* = 8) Improvement (*p* = .71, t = −0.38)

**Table 4.** K10 scores compared at different time points using pairwise t-tests.

	3 months	6 months	12 months
Pre-course	Mean decreased from 23.1 to 22.4 (*n* = 45) Improvement (*p* = .33, t = 0.98)	Mean increased from 22.5 to 22.7 (*n* = 38) Decline (*p* = .87, t = −0.16)	Mean decreased from 19.1 to 17.9 (*n* = 8) Improvement (*p* = .22, t = 1.36)

**Table 5.** SF12 total scores compared at different time points using pairwise t-tests.

	3 months	6 months	12 months
Pre-course	Mean increased from 31.1 to 31.6 (*n* = 45) Improvement (*p* = .52, t = −0.65)	Mean increased from 31.2 to 31.6 (*n* = 38) Improvement (*p* = .63, t = −0.49)	Mean increased from 33.3 to 35.5 (*n* = 8) Improvement (*p* = .31, t = −1.09)

**Table 6.** Brief resilience scale scores compared at different time points using pairwise t-tests.

	3 months	6 months	12 months
Pre-course	Mean increased from 3.1 to 3.2 (*n* = 45) Improvement (*p* = .22, t = −1.24)	Mean increased from 3.15 to 3.21 (*n* = 37) Improvement (*p* = .54, t = −0.62)	Mean increased from 3.5 to 3.6 (*n* = 8) Improvement (*p* = .62, t = −0.53)

**Table 7.** Short Warwick-Edinburgh mental wellbeing scale scores compared at different time points using pairwise t-tests.

	3 months	6 months	12 months
Pre-course	Mean decreased from 22.6 to 22.2 (*n* = 45) Decline (*p* = .42, t = 0.81)	Mean decreased from 22.4 to 22.3 (*n* = 38) Decline (*p* = .96, t = 0.05)	Mean decreased from 25.4 to 24.3 (*n* = 8) Decline (*p* = .41, t = 0.88)

A positive impact from the courses was also seen on the carers’ quality of life. There was a significant improvement when comparing the pre-course scores to the scores after 3 months, with an improvement (higher score) still being seen after 6 and 12 months (Table 3). It should also be borne in mind that due to the progressive nature of dementia, a decline in the carers’ quality of life would be expected in many cases.

Although no significant differences were seen, carers’ psychological distress improved (lower score) following the courses, with the improvement being sustained over the long-term (Table 4).

As for the psychological distress scores, no significant changes were seen for the carer health scores (Table 5). However, there was an overall improvement (higher score) compared to the pre-course scores which was again maintained over time to some extent.

The resilience scores followed a similar pattern to other carer outcomes, with a non-significant improvement (higher score) being seen compared to pre-course scores, and some degree of improvement being sustained over the longer term (Table 6).

The results relating to mental wellbeing show the opposite picture to the other outcome measures as there appears to be a very slight decline (lower score) (Table 7). Even at 12 months, the average mental wellbeing for the eight carers who completed the measures at this time point remained lower than levels seen prior to attending the course.

### Themes from the interviews

**Figure 1.**
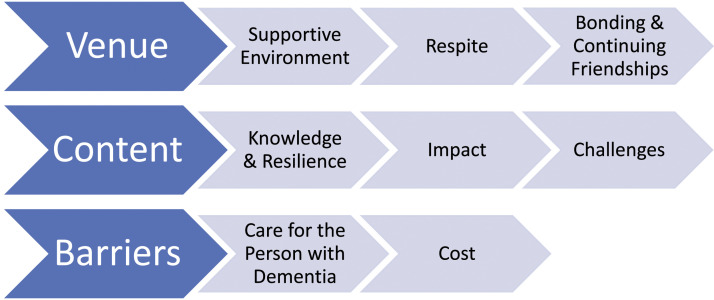
Outcome themes from carer interviews.

Thematic analysis of the carer interviews considered the outcomes for those who attended any of the aforementioned courses offered by DCC. Three overarching themes were identified, together with a variety of sub-themes as indicated below (Figure 1).

### Venue

This theme considers venue in terms of its functionality for carers by exploring the ways in which the residential course and its delivery impacted upon their experience.

#### Supportive environment

Training facilitators were mindful to create an environment where carers felt safe and valued, and carers reflected that this had been achieved:

‘The environment was so supportive…I knew I could ask anything, say anything and no one would throw their hands up in horror.’ (Young Onset Dementia course)

An element of the supportive environment was achieved through the benefit of the course being residential, which meant carers could absorb and immerse themselves in the learning:

‘I think it made a lot of difference…I think if I’d gone home every night I would have missed out a lot.’ (Me, You, Dementia Too course)

Being residential, the carers and facilitators spent a lot of time together, but this was felt to work well from the carer perspective:

‘The whole sort of programme…there was no separation; there was no kind of them and us. It was really kind of embodied in the whole experience.’ (Core course)

#### Respite for carers

Respite through the course being residential was a serendipitous outcome for carers:

‘Just getting away from home…was the real highlight I think because I didn’t think that was possible…to actually be able to stay somewhere else.’ (Young Onset Dementia course)

‘The courses are really good but what was nice was actually having the time away…so it’s a bit of a luxury…it makes a big difference.’ (Core course)

#### Bonding and continuing friendships

Having a residential venue afforded carers the opportunity to spend extended time with people who have similar caring experiences:

‘Being residential, we were able to meet other people who were in a similar circumstance…it was very empowering to hear other people’s perspectives.’ (Core course)

There was an opportunity for friendships to be forged which can provide alliances and support for the future. Many of the carers continued to remain in contact following the course through various social media or email:

‘We’ve been in touch ever since…that’s been a highlight to be honest…you can still be in contact with people who you’ve actually built that trusting relationship with.’ (Core course)

### Content

#### Knowledge and resilience

Carers valued the content of the course and the knowledge this provided. As one carer noted:

‘No one prepares you to be a carer; you’re in a new game and you don’t know the rules.’ (Young Onset Dementia course)

They also appreciated and benefitted from the way in which the content was delivered:

‘The people that were doing the instruction knew what they were talking about, that filled me with confidence. The content of the course for me was spot on and how it was conducted, I don’t know what you’d call it, but there was interaction.’ (Young Onset Dementia course)

‘There are pieces of information that they’re going to give that will frighten people but they seemed as a group to be able to unravel that fear and take time out with people…that’s where the strength is.’ (Me, You, Dementia Too course)

#### Impact on carer confidence

The skills, knowledge and strategies gained through the course enabled carers to feel more confident in themselves. The carers had the opportunity to benefit from some self-reflection and understand the need to prioritise their own health needs:

‘It gave me more insight…as a result of going on the programme I’ve pushed for some counselling for myself…the course highlighted to me that I wasn’t alone.’ (Me, You, Dementia Too course)

‘I have made a decision to do more self-care…I was putting my mother before me all the time…when I rang up the dentist they told me I hadn’t been for over 2 years, same with the eye test...whereas I’ve made sure mum has seen a dentist and an optician every year.’ (Home to Home course)

The change was also noticed by the training facilitators:

‘You see people arriving on the first day looking very anxious, looking a bit downtrodden, a bit exhausted, a bit worried and by day three, they look different – there’s just something about them; there’s more sparkle about them, they seem more confident, they look more confident, they’re walking more confidently.’

#### Impact on their caring role

There were many ways in which the carers could illustrate how the course content had impacted on their ability to care for their loved one. Carers saw benefits for the person with dementia which resulted from doing things differently:

‘I feel my interactions with my mum are more empowering for her…I don’t try and do everything for her.’ (Core course)

Improving their knowledge of dementia and understanding how this affects a person also helped carers to adjust their expectations:

‘It’s made us realise his perspective a bit better…just understanding how the disease affects them and not letting it get to you anymore.’ (Core course)

Carers were also able to reflect on how a change in their own wellbeing would have an impact on the person they were caring for:

‘When I’m in burnout, he can sense it; when I come out of burn out, I take him with me.’ (Young Onset Dementia course)

#### Challenges encountered

Any learning trajectory will have inherent challenges for learners and these courses were no exception. This was mainly expressed in relation to the emotional nature of the course content and the extent to which carers felt overloaded.

‘I thought that each part was pretty interesting so if anything it was like too much, trying to cram it in, it felt like it was galloping through stuff sometimes.’ (Home to Home course)

‘It felt quite intense…it would have been nice to have time to catch up somehow.’ (Core course)

Although carers enjoyed and appreciated the courses, individual circumstances meant that not all content was relevant to everyone and some areas of interest could be missed. This was particularly noticeable in the early days of delivery where participants attended the core course rather than the bespoke courses which evolved later as a result of carer feedback.

‘I think all our circumstances were quite different…maybe sort of tailoring future courses for those things…people who are sort of at early stage.’ (Core course)

‘The later stages…it wasn’t really gone into too much.’ (Core course)

To some extent, the courses could be seen as a victim of their own success, with carers valuing them so much that they were concerned how they could receive a similar level of support in the future.

‘It was such a good course, but where was I going to get the support once it had finished?’ (Core course)

### Barriers

#### Caring for the person with dementia in the absence of the carer

The courses were designed to meet the needs of carers who support a person living with dementia. The very nature of this relationship introduces a challenge in finding support for that person whilst the carer is away from the home. This challenge is not only expressed in financial terms but was also experienced in relation to *who* would be able to step into that caring role whilst the main carer attended the course.

‘The only barrier would have been if I hadn’t been able to get someone to have my dad.’ (Core course)

‘My children, grown up children, have not done it before, I mean, not overnight anyway.’ (Young Onset Dementia course)

The ‘Me, You, Dementia Too’ course did not have the same issue as carers were able to take the person with dementia with them, but the logistics for this course were not without challenges:

‘I felt to some extent that the total separation of us was difficult for him. The level of activities that have been designed for the dementia sufferers were too low a level and he got quite worried. So I was in a position where I was in a room and could see him wandering round the gardens.’ (Me, You, Dementia Too course)

‘My wife has posterior cortical atrophy...she can’t read or play games so most of what they brought with them to do were things she couldn’t really engage with.’ (Me, You, Dementia Too course)

#### Cost of the courses

Courses during the evaluation were provided free of charge and a number of funding options were explored by DCC including payment for the course or discretionary donation. When asked whether they would be willing to pay for such a course, six carers indicated that they would have had no difficulties paying or being willing to pay for the course. Four carers said they would have been unable to pay with one commenting:

‘No, not at that price. Definitely not. Whilst I understand it’s very costly, at that point it would have been a struggle to be honest.’ (Core course)

Eight carers were unsure. For two, it would be dependent upon the actual charge levied and the ability for them *and* other family carers to attend:

‘Depends on price. Might not have been able to afford for us both to go and my daughter cares for her dad as well.’ (Core course)

For other carers, their concerns about cost were based on the fact that they were not sure the course would be worth the cost *prior* to having attended:

‘I think it was only going on the course that I realised how good it was and how much it really benefitted me. I am not sure that I would have taken a punt beforehand if I had to pay for it basically. I think, sort of, probably testimonials from one of the people because I know when I sort of came back and was telling people about the course and I was sort of saying that, you know, it was free but had it been like £250 and in fact had it been more, I probably would have paid having gone because I felt that my experience for me was so positive.’ (Core course)

‘I probably wouldn’t have gone, however, I’d have missed something really valuable. I think that especially when you do have to pay, or at least make a contribution, it really needs to be sold better. So you need to understand what you’ll get out of it before you go. And that way, you won’t put people off’. (Core course)

## Discussion

Overall, both the quantitative and qualitative analyses demonstrate that attending the DCC Carers Support Courses had a positive impact on carers. Almost every outcome measure saw an improvement that was maintained to some extent over time, indicating that carers generally remained in a better physical, mental and emotional situation than that experienced before the course. This is important insofar as mitigating the negative impacts of caring through improved knowledge and increased confidence can lead to greater positive experiences of the caregiver role (Leocadie et al., 2018). The only difference was carers’ mental wellbeing which declined slightly following the course. It should, however, be noted that the outcome measures relied on carers self-reporting, so results may be influenced by how a carer was feeling at the particular time they completed the outcome measures booklet. It can be expected that carers may have more challenges in their caring role as the dementia develops over time in the person they care for or the impact of caring becomes more burdensome for some.

Improvements were generally maintained over time to some extent, although the decreasing number of returns at each time point was likely to have been a factor in the degree of improvement and lack of statistical significance. In particular, the low number of returns after 12 months makes it difficult to have full confidence in the longer-term impact. Although it could be argued that the use of pairwise t-tests exacerbated this issue by excluding carers if they did not have valid scores at both relevant time points, this only affected a very small number of returns in practice. The choice of analysis also enabled a like-for-like comparison for carers at different time points, rather than comparing a small group of carers after 12 months to a big group pre-course. Although this could potentially have shown a statistically significant change, it would be difficult to know whether this was due to the course or the particular characteristics of the 12-month group of carers. To mitigate for the limitations encountered due to the decreasing returns, the quantitative and qualitative data should therefore be considered together.

Thematic analysis from the carers’ interviews reinforced that the courses helped to enhance carer confidence by improving their knowledge and skills, with carers benefitting from feeling looked after and supported throughout the courses. As Steenfeldt, Aagarup, Jacobsen and Skjodt (2021) note ‘Family caregivers need to learn new skills and knowledge to be able to manage to adapt to their new situation’ (p. 10). They continue to observe from their findings that there is a need for various forms of support and tailored information (*ibid*). The carers also learned how to look after themselves and increase their own resilience, making them feel better able to care for the person living with dementia that they cared for. This subtle shift from relative to caregiver was noted by Quinn et al. (2015) reflecting that ‘caregiving meant that they were able to meet the needs of their relative and ensure the continuation of their relationship with their relative’ (p. 231). This is an important feature for carers who need to manage the complex dynamics of an evolving and changing landscape. Anecdotal evidence suggests that carers have put new ideas and strategies into practice following the courses.

The interviews also demonstrated that the DCC training facilitators have combined three key components to create a unique approach to delivering training to carers. The importance of this lies not only in the information given but the manner in which this is delivered. Steenfeldt et al. (2021) note that ‘a key factor for benefiting from the information appears to be that it is provided in a way that helps the family caregiver feeling competent and in control of the situation’ (p. 10). Course facilitators achieved this in three distinct ways:

Firstly, the approach is fundamentally underpinned by a unique combination of skills harnessed within the training team. As a group of professionals, this team are able to support an emotionally fragile group of people, making them feel safe and supported. They are able to do this in a person-centred way, responding at the individual level.

Secondly, carers had faith in the knowledge, expertise and strengths of the training facilitators. Working from this basis of trust, empathy and respect, the trainers enabled carers to reflect, express themselves, learn from each other and create a community of support.

Thirdly, the residential component was instrumental in nurturing these delicate and complex relationships. From these strong foundations, an educational component was introduced which afforded carers the opportunity to focus on themselves; feeling safe and not alone. They were able to safely share their experiences with others in a similar or relatable situation and often create sustainable friendships.

## Conclusion

There is a paucity of quality courses available to informal carers to enhance the deeper learning of caring for a person with dementia. Although it remains true that every care experience will be as unique as the person cared for, there are lessons that can be learned which will improve the quality of life for the informal carer and support them on their caring journey. Evidence gained from this evaluation demonstrates the impact of the courses offered by Dementia Carers Count and adds to the current weak evidence base relating to dementia courses aimed at preparing carers to care.
